# Health-related quality of life among 13–14 year old adolescents with overweight−a mixed methods approach

**DOI:** 10.1186/s12955-020-01413-0

**Published:** 2020-05-29

**Authors:** T. K. B. Sundar, K. Riiser, M. C. Småstuen, R. Opheim, K. Løndal, K. Glavin, S. Helseth

**Affiliations:** 1 Department of Nursing and Health Promotion, Faculty of Health Sciences, Oslo Metropolitan University, P.O. Box 4, St. Olavs plass, NO-0130 Oslo, Norway; 2 Department of Nursing Science, Institute of Health and Society, University of Oslo, P. O. Box 1130, Blindern, N-0318 Oslo, Norway; 3 Department of Physiotherapy, Faculty of Health Sciences, Oslo Metropolitan University, P.O. Box 4, St. Olavs plass, N-0130 Oslo, Norway; 4 Department of Primary and Secondary Teacher Education, Faculty of Education and International Studies, Oslo Metropolitan University, P.O. Box 4, St. Olavs plass, N-0130 Oslo, Norway; 5 VID Specialized University, Faculty of Health Studies, P.O. Box 184, Vinderen, NO-0319 Oslo, Norway; 6 Faculty of Health Sciences, Oslo Metropolitan University, P.O. Box 4, St. Olavs plass, N-0130 Oslo, Norway

**Keywords:** Health-related quality of life, Adolescents, Overweight, Obesity, Physical activity, Mixed methods

## Abstract

**Background:**

Overweight and obesity are public concerns with risk of adverse health outcomes. Health-related quality of life (HRQoL) is lower in adolescents than children in general. An increase in body mass index (BMI) is associated with a decrease in HRQoL. The purpose of this study was to measure and explore the HRQoL among adolescents with overweight or obesity who had participated in an intervention study, Young & Active, with the aim of increasing physical activity (PA), reducing BMI and promoting HRQoL.

**Methods:**

Mixed methods, with a convergent design, were used to investigate how different methodological approaches could expand our understanding of the adolescents’ HRQoL. Quantitative post-intervention data on HRQoL were collected among the 84 intervention participants, aged 13–14 years, using the KIDSCREEN 52 questionnaire. The data were compared with a Norwegian reference population of 244 individuals, and analysed using a non-parametric Mann-Whitney test. Qualitative semi-structured interviews were conducted with 21 adolescents from the intervention. A directed approach to content analysis was adopted, using the ten sub-scales from KIDSCREEN 52.

**Results:**

HRQoL in the intervention sample was significantly reduced on the sub-scale of physical well-being compared to the reference population. The reference population scored significantly lower than the intervention sample on the sub-scale of parent relation and home life. No significant differences were found on the other sub-scales. The qualitative data supported the quantitative findings on the sub-scale of physical well-being, but showed that perceptions of fitness, energy level or health could vary. Regarding parent relations, the interviewees extended this to include relationships to other family members as equally important. Most of the interviewees expressed a negative view of their bodies, but not their clothing or accessories. This may explain why no statistically significant differences were found on these aspects in the results from the KIDSCREEN questionnaire.

**Conclusion:**

The use of the KIDSCREEN 52 instrument gave important indications about the adolescents’ HRQoL and need for additional follow up. The qualitative data provided an in-depth understanding that nuanced the findings and widened our knowledge of the adolescents HRQoL. Combining methods enabled a comprehensive approach to research on HRQoL.

## Introduction

Overweight and obesity are public health concerns globally. Extensive research has investigated the medical challenges associated with these conditions. Growing evidence indicates that overweight or obesity may impact negatively on well-being and functional health [1, 2].

Physical activity (PA) during childhood is associated with a multitude of health benefits due to its preventive effects on several physical conditions and its ability to stimulate cognitive performance and mental well-being [3]. A review article and a meta-analysis provide evidence that adolescents who are overweight or obese are less physically active and less physically fit than their leaner counterparts [4, 5]. While physical activity is found to be positively related to HRQoL, sedentary behaviour, on the other hand, is found to be negatively related [6]. A closer look at a combination of Body Mass Index (BMI) and PA among adults indicates that PA is important for HRQoL regardless of weight status [7]. Research shows that the association between PA, body composition and fitness on HRQoL among children has received less attention [7, 8]. Little is known about other factors that may contribute to the results, such as functional difficulties due to overweight, or psychosocial mediators [9].

HRQoL is a multidimensional construct for assessing subjective perspectives of psychosocial, physical and functional components of well-being and health as perceived by individuals [10]. The use of instruments for measuring HRQoL may help identify people at risk of developing health problems, or assist in determining the burden of a health problem, such as overweight or obesity [11]. In the last decade, research reflecting children’s views has become increasingly important [12]. From 2001 to 2004, KIDSCREEN, a multidimensional generic questionnaire, was developed and tested in Europe as a collaborative project between 13 countries. The KIDSCREEN instrument comprises three versions for children, KIDSCREEN 52, KIDCREEN 27 and KIDSCREEN 10, as well as a parent proxy version. The content of the questionnaires is based on literature reviews, focus group interviews with children and parents, and opinions from experts on HRQoL measurements for children. The instruments aim to capture HRQoL from the perspectives of both healthy and chronically ill children by assessing their subjective health and well-being [13].

Adolescence is considered a critical period in life, characterized by physical, psychological and social changes. Most adolescents go through this period without any trauma. However, studies report that in the general population, adolescents rate life satisfaction and health-related quality of life (HRQoL) lower than children, and girls lower than boys [14, 15]. Research shows that this is further aggravated by increased Body Mass Index (BMI) [16, 17].

Recent research shows that self-determination theory (SDT) is useful for understanding perspectives on adolescents’ well-being and quality of life [18]. SDT postulates that subjective well-being or feeling positive and being satisfied with life is determined by satisfying the three basic psychological needs; autonomy, competence and relatedness, and that social environments that promote such needs provide optimal conditions for an individual’s development, well-being and motivation [19]. Motivational Interviewing (MI) is a collaborative, person-centred form of counselling approach that can be understood in terms of SDT. The method has shown to be effective in strengthening motivation for change [20].

The specific aim of this study was to measure and explore the HRQoL among adolescents with overweight or obesity who had participated in an intervention study, Young & Active (ClinicalTrials.gov NCT01700309). The overall aim of the Young & Active intervention study was to increase PA, reduce BMI and promote HRQoL, through the use of principles from SDT and MI [21]. As part of Young & Active, qualitative interviews were conducted with a sample of adolescents from the intervention. The purpose of the interviews was to gain an in-depth understanding on how the intervention had influenced the adolescents’ quality of life and everyday lives.

Mixed methods were chosen to investigate how different methodological approaches could expand our understanding of the adolescents’ HRQoL.

The research questions were as follows:

1. How do the participants in the Young & Active intervention group respond on the KIDSCREEN questionnaire compared to the Norwegian survey reference population?
2. How do the Young & Active participants describe their health-related quality of life in the interviews?

## Methods

### Mixed methods

We used a mixed methods approach with a convergent design. The quantitative and qualitative data were collected in parallel during the same time frame by the research group behind this article [22]. A subsample of interviewees was recruited from the Young & Active population that completed the KIDSCREEN 52 survey [23, 24]. The quantitative and qualitative sets of data were analysed separately, and then brought together for analysis and comparison during the discussion.

### Study context and participants

The 12-week intervention study, Young & Active, was conducted with adolescents, age 13–14, from 2012 to 2014. School nurses in three counties from rural and urban districts of south-eastern Norway assisted in the recruitment to Young & Active. Following the recommended height and weight measurements in the 8th year [25], adolescents with age and gender adjusted BMI above 25 were invited to participate in the intervention. Adolescents involved in treatment programmes or other interventions were not eligible. A total of 84 adolescents volunteered to participate. As part of the Young & Active study, measurements of height and weight, a standard fitness test, and self-report instruments were individually completed at the adolescents’ schools. During the intervention, the adolescents were asked to register all weekly PA. This allowed them to follow their activity level on graphs and figures. They also kept a PA diary and could ask questions about PA if they wished. Once a week, they received online feedback and counselling from one of the researchers, an experienced physiotherapist. The counselling was based on principles from Motivational Interviewing (MI). Knowing that overweight or obesity, and adolescence in itself, potentially increases adolescents’ vulnerability, we considered it relevant to complement the study with qualitative interviews of a sample of the participants, following the post-intervention measurements and testing in the Young & Active intervention study [9, 12, 21, 26].

The qualitative data involved semi-structured interviews with a sample of 21 adolescents selected among the 84 participants in the Young & Active intervention. They were recruited to interviews by the researchers during the testing shortly after completion of the intervention. All adolescents were asked if they wanted to participate in the interviews. Based on a strategy of convenience sampling [27], we accepted those who said yes to our request. Seven boys and 14 girls agreed to be interviewed. Distribution of gender, weight and cardiorespiratory fitness among the interviewees followed the main study [28]. After the interview sessions, we conducted a review of the data to consider if more informants were needed [29]. We concluded that 21 interviews was sufficient and that saturation was obtained, meaning no new themes were emerging [30].

In addition, data from a larger Norwegian HRQoL validation study involving 244 individuals, age 13–14, were applied. These data were extracted from a cross-sectional study, conducted across 20 randomly selected schools in Norway, involving 1123 children, age 8–18 [31].

### Measurement of health-related quality of life

HRQoL, in the Young & Active study, was measured using the Norwegian version of the KIDSCREEN-52 questionnaire [31]. The questionnaire covers ten subscales of social, mental and physical well-being, with five to seven sub-items rated by each individual using a five-point Likert scale [13, 32]. The scale indicates the frequency of certain behaviours or feelings (1 = never to 5 = always), or the intensity of an attitude (1 = not at all to 5 = extremely). The time frame refers to the previous week. The number of items in each dimension is illustrated in Table 1. The dimension scores in KIDSCREEN were converted into a 0–100-point scale, with 100 indicating the best HRQoL and 0 the worst. The KIDSCREEN instrument has been tested on 22,827 children aged 8–18 years across Europe and has shown satisfactory reliability and validity, both on healthy children and children with chronic health conditions [34]. In Norway, the instrument was tested and validated on 1123 children, aged 8–18 years, showing a Cronbach’s alpha value above 0.80 for all KIDSCREEN scales, which suggests good internal consistency reliability for the instrument [12, 13, 31].

**Table 1:** Overview of KIDSCREEN-52, a short description [33]

	KIDSCREEN scales	No. of items	Short descriptions
1.	Physical well-being	5	Level of physical activity, energy, fitness and health
2.	Psychological well-being	6	Positive and negative emotions, life satisfaction and optimism
3.	Mood	7	Degree of depressive moods and stressful feelings
4.	Self-perception	5	Positive or negative bodily appearance, through questions about satisfaction with looks, clothes and accessories
5.	Autonomy	5	Freedom of choice and opportunities to create social and leisure time
6.	Parent relation and home life	6	Relationships with parents and home atmosphere
7.	Financial resources	3	Perceived quality of financial resources
8.	Social support and peers	6	Social relations with other adolescents
9.	School environment	6	Perceptions of cognitive capacity, feelings about school
10.	Social acceptance (bullying)	3	Aspects of feeling rejected by peers in school

### Statistical analysis

We used IBM SPSS version 25 to conduct statistical analyses of the KIDSCREEN data. Continuous data were not normally distributed and were therefore described using median and range. Categorical data were presented as counts and percentages.

As the data did not follow normal distribution, non-parametric Mann-Whitney tests were used to examine the differences between the Young & Active sample and the reference sample on the ten dimensions of the KIDSCREEN scales.

In order to achieve an accurate estimate, bootstrapping was applied to the sample. When a sample is relatively small, this is a useful method of increasing the sample size without having to collect repeated samples from the population of interest [35]. *P*-values < 0.05 were considered statistically significant and all tests were two-sided. As the study was considered exploratory, no correction for multiple testing was performed.

### Qualitative interviews

The research group developed a semi-structured interview guide containing 11 open-ended questions (see Table 2). The interview guide ensured consistency and flexibility in the approach to enable the adolescents’ accounts to emerge. Probes were open and specific to the adolescents’ comments. The interview guide included questions that focused on the adolescents’ everyday lives. Examples of questions included; could you describe yourself to me, your everyday life, health, sleep, self-image, body image, life satisfaction, moods and emotions, family relations, leisure time activities, friendships, school, views on PA and perceptions of participation in Young & Active. The first author conducted all 21 individual interviews following the post-intervention measurements in Young & Active. Interviews took place during school hours at the adolescents’ schools with only the adolescent and the interviewer present. In our opinion all adolescents spoke freely, but some of them were less talkative than others and gave shorter responses. The longest interview took 70 min. All interviews were audio-recorded and transcribed verbatim by the first author.

**Table 2:** Overview of questions in the semi-structured interview guide

**1. Family and everyday life**	**2. Friends**	**3. School**
Whom do you live together with? What does a typical day look like for you? What do you like to do in your spare time, and together with whom? Meal patterns at home, and in school?	Do you have friends? How often do you meet with friends, and what do you do together?	How do you experience school (courses, teachers, classmates)? Do you participate in physical education classes?
**4. Self-image**	**5. Mood**	**6. Health**
What do you think about yourself? What do you think others think about you?	How would you describe your mood? What is important for you to feel satisfied in your everyday life?	What are your thoughts about health? How do you experience your own health?
**7. Sleep**	**8. Activities**	**9. Physical activity**
Do you sleep well? What is good sleep for you?	What kind of activities do you enjoy doing? Is there anything that prevents you from participating in those activities?	Could you tell me your thoughts about physical activity? What motivates you to be physically active? Is there anything that hinders you from being physically active?
**10. Organized sports**	**11. Experiences with participation in the research project**	
Do you participate in any organized sports? How often? How do you experience participation? Do you participate for your own enjoyment?	What made you join the Young & Active intervention? Was anyone other than you involved in the decision? How did you experience the intervention?	

### Qualitative content analysis

The interview data were analysed using a qualitative directed content analysis [36]. The ten KIDSCREEN subscales were used as key concepts and theoretical basis (see Table 1).

The first author listened to the recordings and read through the transcripts multiple times, and coded the adolescents’ statements according to the ten subscales and definitions in KIDSCREEN 52. All statements were then summarized. The results were discussed and agreed upon in the research group.

### Ethical considerations

The study was approved by the Norwegian Regional Committee for Medical and Health Research Ethics (REK no. 2010/2978A). Parents gave their written consent for their children to participate in the Young & Active intervention study and in the qualitative research interviews after completion of the intervention. The adolescents also gave their written assent. Information about the study, confidentiality, potential hazards and benefits, the purpose of the study and the right to withdraw were emphasised, both in the written material and verbally to the adolescents. The anonymity and confidentiality of the participants and the material were ensured by using subject identifier codes instead of names. School nurses were aware of the study and were prepared to help adolescents who needed support in cases of personal difficulties.

## Results

### HRQoL data

In total, we analysed KIDSCREEN data on 84 individuals from the intervention sample and 244 from the Norwegian reference sample. The proportion of girls compared to boys was higher in both samples (61 and 58% for Young & Active and the reference population, respectively). Statistically, findings from the Young & Active sample showed a significantly lower HRQoL on the subscale of physical well-being. The Norwegian reference population had a statistically significant lower HRQoL on the parent relations dimension (see Table 3).

**Table 3:** Comparison of median scores between study sample and reference sample on Kidscreen-52 domains

	Young & Active			Reference			
	N	Median	95% CI	N	Median	95% CI	*P*- value
Physical Well-being	67	55.00	50.00–60.00	244	70.00	67.50–70.00	**< 0.01**
Psychological Well-being	72	77.09	70.83–85.42	244	79.17	75.00–79.17	0.85
Mood and Emotions	75	82.14	78.57–89.29	244	85.71	82.14–85.71	0.81
Self-Perception	72	62.50	47.50–75.00	244	70.00	67.50–75.00	0.46
Autonomy	70	80.00	70.00–87.50	244	70.00	70.00–75.00	0.10
Parent Relation and Home Life	70	83.33	75.00–91.67	244	75.00	75.00–79.17	**< 0.05**
Financial Resources	72	100.00	87.50–100.0	244	83.33	75.00–87.50	0.06
Social Support and Peers	70	83.33	77.09–87.50	244	70.83	68.75–75.00	0.19
School Environment	71	68.75	62.50–75.00	244	62.50	62.50–66.67	0.25
Social Acceptance (Bullying)	74	100.00	91.67–100.0	244	91.67	91.67–100.00	0.25

Further, all analyses were stratified by gender (data not shown in table). The subscale of physical well-being showed that both boys and girls from the Young & Active sample reported a statistically significantly lower HRQoL for this subscale compared to the reference population, with values ranging from 45.00–60.00 for girls and from 50.00–70.00 for boys. On the subscale of parent relations, the girls from the reference sample had statistically a significantly lower HRQoL score compared to the girls from the Young & Active sample, ranging from 70.83–81.20 in the reference sample and from 79.17–91.67 for Young & Active. Among the boys there were no statistically significant differences between the groups on this subscale. No other statistically significant differences were found for any of the remaining subscales.

### Qualitative data

There were qualitative findings on all ten subscales, although the findings on the subscales of Physical Well-being and Self-Perception were the most prominent and will be treated in more detail than the other qualitative data. The findings in the remaining subscales will be presented in a summary. Quotes from the interviewees used in the text have been translated from Norwegian.

### Statements about physical well-being

Most of the adolescents stated that they felt increasingly fit and motivated to engage in PA after participating in Young & Active. However, they still described their health, fitness and energy level as being below average or lower than their peers. For example, one girl said: *I’ve always struggled with physical activity because I’m not the strongest or the fastest person. I think my fitness level has increased but I haven’t noticed that much change (Inf. 10).* When talking about their health, many adolescents expressed that their health was poor. They attributed this to weighing too much, not being active enough or liking food too much. A few talked about having health problems such as asthma, migraine, or complaints regarding neck, knees or heels. Other adolescents presumed that their health was ok, since they were not ill. Several adolescents emphasized that the extent to which they felt energetic, fit and healthy could vary significantly from week to week or even from day to day, depending on other factors such as amount of sleep, food intake, pain or disease – as one boy said: *My health differs from day to day*. *Sometimes I feel less energetic, perhaps because I went to bed too late (Inf. 18).* For some adolescents, feeling energetic was associated with the weather. One girl said: *I feel I can have loads of energy if the weather’s nice, then I can go out and run around. But if it’s raining, I’ll lie on the sofa and play computer games (Inf. 5).* When enjoying and mastering a physical activity, even pain seemed to be less significant, as one boy stated: *I have some heel problems. I don’t notice it when I’m slalom skiing and slalom skiing is my favourite activity (Inf. 1)*. Other adolescents expressed similar opinions and stated that the extent to which they became involved in physical activities or felt energetic depended on their feelings about the physical activity they were thinking about engaging in, as this girl stated: *I guess I have the energy, unless I don’t feel like it, and then I can be quite lazy (Inf. 4).* This girl was regularly active in a sport that she enjoyed, had chosen by herself and which she mastered. When discussing other types of PA, she described herself as a person who didn’t like moving. Their feelings about PA also related to exercising together with friends, as this boy stated: *I prefer to exercise with my friends because we push each other. Then I feel I’m getting more out of the exercise and enjoy it (Inf. 6).*

### Statements about self-perception

Most of the adolescents appeared to have a negative view of their bodies, as this girl stated: *I’m not happy with my body. I feel I’ve grown too fast and that other people look at me. The problem is that all my friends are so thin, and here’s me with my large thighs and fat belly. I consider myself fat (Inf. 7).* Another girl said that she often envied thin girls and wished she could have been like them: *Sometimes I used to think, I want to look like her, and she’s so thin and beautiful (Inf. 2).* For some, thoughts about weight were coupled with their perceptions of self, as this girl explained: *I don’t think too highly of myself. It’s a bit hard because I’m not used to talking about it, but I’m not happy about myself because I actually think that I weigh too much (Inf. 5).* Most of the boys appeared to be more preoccupied with building muscles than getting thin, as this boy stated: *I like bigger muscles; they make me feel good (Inf. 1). I’m not that attractive yet, but I will be when I’ve done some more training (Inf. 17).* Some of the boys admitted that the main reason they exercised was to lose weight, as this boy stated: *I think a lot about my weight and that’s the main reason why I do training (Inf. 3).* However, like the other boys, he also wanted to achieve weight loss through training and building muscles. A number of the adolescents appeared to distinguish between negative thoughts about their bodies and how they viewed themselves as persons, as one girl said: *I’m sort of satisfied with myself, but not with my body. I want to change my looks and lose weight. But I think my personality is ok (Inf. 11).* The adolescents’ views about themselves were also associated with thoughts about how their friends viewed them, as this boy stated: *I’m a happy person who makes friends easily. I’m satisfied with myself and I think other people like me and regard me as a kind and caring person (Inf. 15).*

### A summary of findings in the remaining subscales

Within the subscales of psychological well-being and mood, many adolescents stated that they felt happy most of the time but emphasised that this could vary considerably. Some of them said that eating too little could make them feel unhappy and irritable. Several of them stated that if their friends and family were happy, then they were happy, and vice versa. Most of them expressed that spending time with friends made them feel happy and put them in a good mood. They also said that good sleep was important for their moods. Some of them stated that they felt generally insecure about themselves, feeling pressure regarding how they should behave, how they should dress and how they should look.

Within the KIDSCREEN subscales of autonomy, parent relations, financial resources and social acceptance, a few adolescents expressed insufficiency, i.e. feeling a lack of freedom of choice, lack of parental support or too restrictive parents, lack of money to pay for sports activities because of their family’s financial situation, or being bullied at school. Most of the adolescents said that they received enough support from their parents and that they had not been bullied. Regarding family relations, they extended this to include siblings and other relatives. Many of them expressed that their feelings were influenced by other family events, such as loss of close family members etc., and not just their direct relationship to their parents.

All the adolescents affirmed that they had friends. Except for a couple of adolescents who told about being bullied at school, the majority said that they were friends with their classmates. All of them stated that they had friends outside school, although for some of them, these were mainly online friends.

Regarding the subscale of school environment, most of the adolescents stated that they liked their schools. The majority said that they felt increased pressure from their parents and teachers about achieving good grades and doing more homework. Several of them expressed that getting bad grades negatively impacted their self-confidence.

## Discussion

We used a mixed methods approach with a convergent design on the post-intervention qualitative and quantitative data [37]. Measuring HRQoL provides important information on how adolescents who are overweight or obese feel about their lives. A survey of HRQoL on 17,159 children across 10 European countries using the generic KIDSCREEN-52 instrument found that overweight or obese children and adolescents had a statistically significant lower mean HRQoL score than children of normal weigh. The scores were lowest in the sub-scales of physical well-being, self-perception, social acceptance and bullying [12]. However, assessing HRQoL through questionnaires is a subjective task, involving cognitive processes that are influenced by biological, psychological and social-environmental factors [38]. Applying both qualitative and quantitative methods provided an opportunity to investigate how the different methodological approaches could complement and nuance each other in understanding the adolescents’ HRQoL [36].

PA is associated with many health benefits, including improved HRQoL and self-esteem [39]. Most of the adolescents who had participated in the Young & Active intervention study were still overweight or obese after participation. Post-intervention KIDSCREEN 52 data from the Young & Active study showed that the adolescents had statistically significant lower scores on the subscale of physical well-being compared to the Norwegian reference population. The data was verified as lower among both genders in the intervention group. Other studies on adolescents who are overweight or obese have shown similar results [1, 2, 10, 12, 40].

The qualitative data analysed according to the KIDSCREEN subscale of physical well-being supported the KIDSCREEN results from the Young & Active sample. However, the data highlighted other important factors. Most of the interviewees said that they felt their level of energy, fitness and physical activity was lower than their peers, but still generally better after they had participated in Young & Active. The results from the Young & Active study indicated that the tailored PA intervention had a beneficial short-term effect on cardiorespiratory fitness, HRQoL and BMI [41]. However, the interviewees stated that their physical well-being could vary considerably from day to day, depending on either external or internal factors, such as weather, daily energy level, health conditions, relations to friends and motivation towards an activity. The adolescents’ comments demonstrated that the ways in which they might answer questionnaires could vary, depending on their subjective feelings at the time. Also, an analysis of the qualitative data revealed that they had varying understandings of concepts such as health. Some of them described their health as being poor due to being overweight. Others state that they were in good health because they had no diseases. Different ways of understanding concepts could lead to misinterpretation of the same items between subgroups or measurement errors resulting from discrepancies in how the questions were answered in the questionnaires [38].

On the KIDSCREEN subscale of parent relations, the girls in the reference population had statistically significantly lower scores than the adolescents in the intervention sample. There was no statistically significant difference between the boys in the two samples. Researchers have suggested that parent-adolescent conflicts peak during early adolescence and that this is a natural part of adolescent development [42, 43]. Research also shows that adolescent girls generally report a lower HRQoL than adolescent boys [44, 45]. This may explain why the girls from the reference population scored lower, although it does not explain the difference between the two groups. Other studies have shown that adolescents with overweight or obesity report a lower HRQoL on their relations to parents and family [46, 47]. The data in this study was post-intervention data. Participation in the intervention may have impacted the adolescents’ responses. Getting permission from their parents, and for some being pushed to participate, may have been experienced as supportive. Furthermore, recruitment to studies always entails a selection of participants and the adolescents’ parents may have been more initially supportive.

The interview data in the subscale of parent relations supported the findings from the intervention study sample, with a few exceptions. Most of the adolescents expressed positive feelings towards their parents but expanded their descriptions to include relations to other family members as being equally important to their well-being. This has also been reported in another qualitative study [17]. Some of the interviewees appeared to feel that they were more controlled by their parents. These adolescents expressed a generally lower HRQoL. Recent studies have found that satisfaction of the basic psychological needs for autonomy, competence and relatedness significantly predicts positive effects on general well-being and on PA within sport contexts [18, 48]. Studies have shown a correlation between more positive relationships with parents and better weight and health-related behaviours among adolescents, as well as improvements in quality of life among those who experience an increase in parent support [49, 50].

No statistically significantly differences were found on the other subscales although the data revealed some dispersion in CI and median between the Young & Active sample and the Norwegian reference sample. The greatest variation was shown on the subscale of self-perception, with the intervention sample showing a CI between 47.50 and 75.00 with a median of 62.50, and the reference sample showing a CI between 67.50 and 75.00 with a median of 70.00. This suggests considerable variation between individuals in the Young & Active sample. KIDSCREEN measurements on this subscale have been found to be lower among adolescents in general compared to children, with decreasing scores among adolescents with overweight and even more so among those who are obese [12, 51]. Research also shows gender differences, with HRQoL among girls declining more than boys during adolescence [45]. In this material, the girls in both groups had lower scores than the boys.

The qualitative data differed from the Young & Active KIDSCREEN results on the subscale of self-perception. Most of the adolescents were concerned about their looks and wanted to change their bodies. However, none of them stated that they were concerned about clothing or other accessories. Recent studies have indicated that adolescents’ body image is influenced by the media and current trends in exercise, diet and beauty, which may contribute to unhealthy body perception and beliefs about the ideal body [52]. Body image has always been an important part of societies and cultures, and overweight or obese individuals are frequently subject to weight-related stigma, either through the media or through bullying [53, 54]. Among adolescents, weight-related stigma is reported to be one of the most prevalent forms of bullying at school [55, 56]. Only a few adolescents in this study stated that they had been bullied. Most of them claimed that they had friends and that they were accepted by their peers. A qualitative study on adolescents’ perception of quality of life underlined the importance that adolescents ascribe to friendship [17]. The adolescents in this study described what could be understood as being a coping strategy by separating views of their personalities from views of their bodies, stating that they liked their personality but not their body. Feeling accepted by friends was an important part of a positive view of own personality. The interviewees still expressed weight-related stigma, probably caused by societal influences. If internalized, such sense of stigma could be even more severe and contribute to a reduced HRQoL, as well as interfere with their efforts to improve their health [57, 58]. The general decline in HRQoL among adolescents in this age group regardless of their weight status, as well as not being dissatisfied with clothing and other accessories, may explain why the intervention KIDSCREEN data did not show any statistically significant difference compared to the reference population on this subscale. However, the qualitative data revealed that the adolescents probably struggled more than what emerged from the quantitative data. Furthermore, they experienced increased pressure from parents and teachers regarding succeeding at school. This may have placed a considerable extra burden on the adolescents’ self-perception and HRQoL.

### Strengths and limitations

The KIDSCREEN data provided important knowledge about HRQoL among adolescents with overweight and obesity that should be taken seriously. Additional qualitative data on the adolescents’ experiences helped to extend knowledge about contributors to resilience among these vulnerable groups. Furthermore, the combined data revealed how different methods may engender complementary results and expand our knowledge [23]. The directed content analysis approach to qualitative interviews does present challenges to the naturalistic paradigm. Using a concept such as KIDSCREEN in the processing of data has some inherent limitations in that the data are approached with an informed, but nonetheless, strong bias. Thus it would be more likely to find evidence that is supportive rather than non-supportive [36]. We were aware of this during the process.

The sample size of the Young & Active intervention is relatively small and may not be representative of a larger population of adolescents with overweight or obesity. Only a sample of 21 participants was interviewed. To avoid initial influence on their views of the Young & Active intervention study, the interviews were conducted post-intervention. Pre- and post-intervention interviews could have provided different results, and so could interviews with all participants in the intervention study. Using other stratifications such as comparing BMI categories, weekly PA level and HRQoL, with the interviewees’ statements, could have strengthened the study. This was considered, but we chose not to do so in order to protect the adolescents’ anonymity.

## Conclusion

The results of HRQoL surveys may provide important indications of the need for additional exploration and follow-up [10]. The qualitative data showed that perceptions of fitness, energy level or health may vary from day to day, based on both external and internal factors. The results also revealed that the adolescents had different understandings of the concepts and may therefore have answered the questions differently. Combining methods enabled a comprehensive approach to researching HRQoL in overweight or obese adolescents. They are vulnerable, specifically in relation to their physical well-being, but also to their self-perception, or at least their body image. Responses regarding self-perception may have provided better results on HRQoL because of the way in which the questions were formulated, integrating clothes and accessories, as well as looks. The qualitative and quantitative data in a mixed methods combination confirm the importance of developing and testing intervention programs that enhance physical well-being and self-perception, using methods that promote resilience and that do not result in adolescents feeling more stress or weight-related stigma. The data underline the importance of supporting the basic psychological needs for autonomy, competence and relatedness for promoting HRQoL. Even though the qualitative findings show that there is a potential risk of misinterpreting the questions, or that other factors may affect the way in which adolescents respond, HRQoL questionnaires such as KIDSCREEN 52 are valuable tools. More research using the benefits of mixed methods approaches may further elucidate our understandings of adolescents as relates to weight and HRQoL.

## Data Availability

The data that support the findings in this study will not be made publicly available, due to participant confidentiality restrictions. Interested investigators may contact the researchers to explore data sharing options.

## Abbreviations

SDT: Self-determination theory
HRQoL: Health related quality of life
MI: Motivational interviewing
PA: Physical activity
BMI: Body mass index
